# Comparison of scar signal quantification using phase corrected and conventional magnitude inversion recovery delayed enhancement imaging in patients with ischemic and non-ischemic cardiomyopathy

**DOI:** 10.1186/1532-429X-15-S1-P190

**Published:** 2013-01-30

**Authors:** John Stirrat, Michael Salerno, David Scholl, Terry Thompson, Maria Drangova, James A White

**Affiliations:** 1 Biomedical Imaging Research Centre, Robarts Research Institute, University of Western Ontario, London, ON, Canada; 2 Medicine, Radiology, and Biomedical Engineering, University of Virginia, Charlottesville, VA, USA; 3 Lawson Health Research Institute, University of Western Ontario, London, ON, Canada; 4 Medical Biophysics and Medical Imaging, University of Western Ontario, London, ON, Canada; 5 Division of Cardiology, Department of Medicine, Schulich School of Medicine and Dentistry, University of Western Ontario, London, ON, Canada

## Background

Myocardial scar volume quantification has been shown to predict response to medical, surgical, and device therapy. Phase sensitive inversion recovery (PSIR)-based Late Gadolinium Enhancement (LGE) image reconstruction is clinically attractive for its reduced dependence on accurate prescription of the Time from Inversion (TI time), and is becoming a preferred approach for many centers. However, while an efficient approach for the visual interpretation of myocardial injury, the influence of this approach on signal-threshold based scar volume quantification has been poorly explored.

## Methods

A total of 80 patients with obvious myocardial scar by LGE imaging (40 ischemic, 40 non-ischemic) underwent blinded evaluations of total scar volume (%LV mass) using matched MIR and PSIR short axis images. Analysis was performed using the Signal Threshold Versus Reference Myocardium (STRM) technique at ≥2, ≥3, and ≥5 SD thresholds. In those with ischemic scar the Full Width at Half Maximum (FWHM) approach was incrementally evaluated. Linear regression and Bland-Altman analyses comparing MIR verses PSIR-based scar quantification was performed.

**Figure 1:**
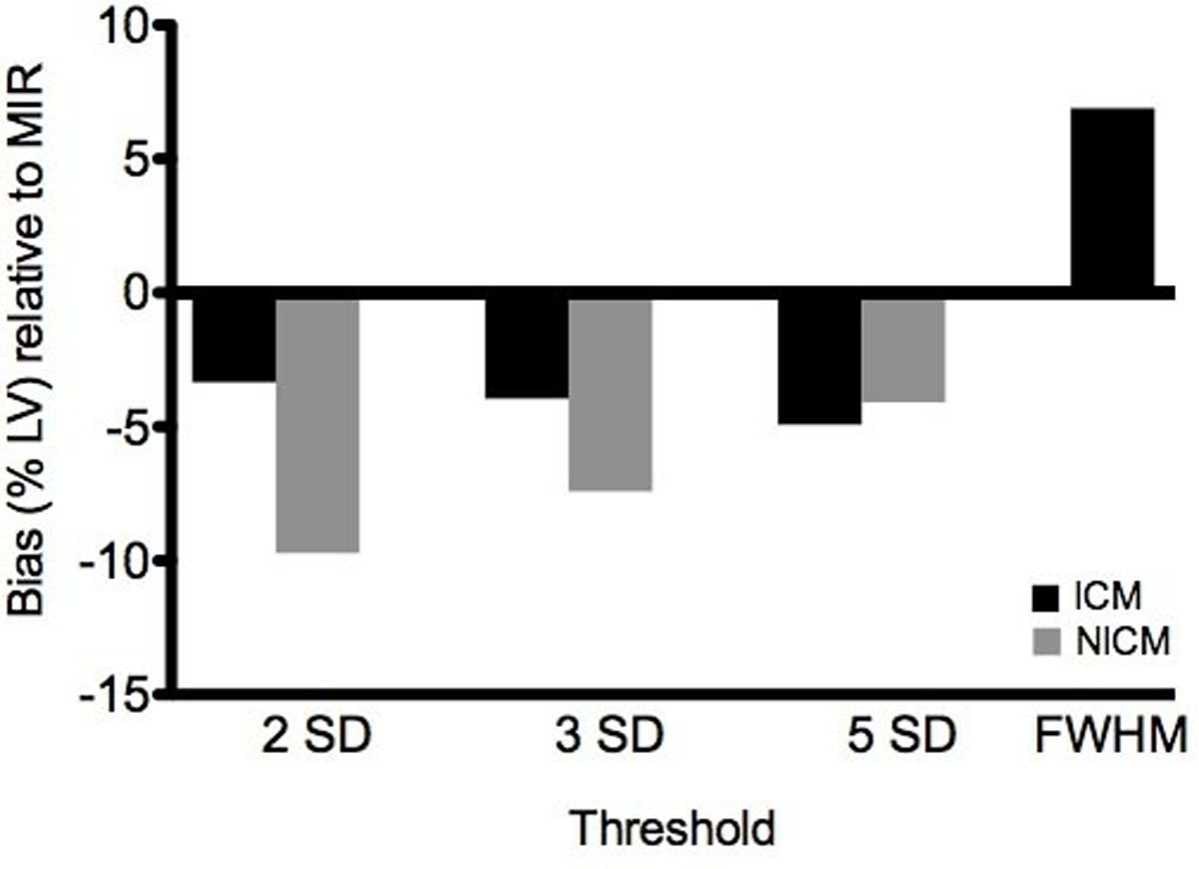
Average bias (&LV) when the PSIR technique is employed (relative to MIR) for patients with ICM (black), and NICM (gray)

## Results

Linear regression analysis demonstrated an excellent correlation between PSIR and MIR-based STRM scar volumes at all 3 STRM-based thresholds for both ischemic scar (r=0.96, 0.95, and 0.88, respectively) and non-ischemic scar (r=0.86, 0.89, 0.90, respectively). FWHM analysis showed good correlation in ischemic scar (r=0.83). Bland-Altman analysis of STRM analysis showed a systematic bias with lower scar volumes produced by PSIR reconstruction images for both ischemic and non-ischemic scar. These differences were modest using STRM for ischemic scar (-3.3, -4.0 and -4.9%, respectively), but greater for non-ischemic scar (-9.7%, -7.4% and -4.1%, respectively). Conversely, ischemic scar analyzed using the FWHM approach on PSIR images produced higher scar volumes than MIR (+6.89%).

## Conclusions

Scar volume measures obtained from PSIR-based LGE images correlate well with MIR-based images. However, a systematic bias exists resulting in reduced volumes being reported for PSIR-based images for STRM analysis, and increased volumes using FWHM analysis. This has important implications for the performance of multi-center clinical trials adopting both PSIR and MIR-based LGE techniques, and raises a potential need to define technique-based scar volume thresholds for prediction of cardiovascular events.

## Funding

Funding for the research was provided in part by the Heart and Stroke Foundation of Ontario grant NA 6488 (to Dr. White) and the Canadian Foundation for Innovation Leaders opportunity fund 18847 (to Dr. White).

